# Incidence, prevalence, and prognostic impact of sarcopenia on hepatic and cardiovascular outcomes in non-cirrhotic metabolic dysfunction–associated steatotic liver disease

**DOI:** 10.3389/fepid.2026.1779600

**Published:** 2026-06-11

**Authors:** Rishi Chowdhary, Manjeet Kumar Goyal, Megh Patel, Rahul Chowdhary, Kirti Arora, Varun Mehta, Akash Batta, Omesh Goyal, Ashita Rukmini Vuthaluru

**Affiliations:** 1 Department of Pediatrics, MetroHealth Medical Center, Case Western Reserve University, Cleveland, OH, United States; 2 Department of Internal Medicine, Cleveland Clinic Akron General, Akron, OH, United States; 3 Department of Internal Medicine, B. J. Medical College, Ahmedabad, India; 4 Department of Internal Medicine, Cleveland Clinic Main Campus, Cleveland, OH, United States; 5 Department of Gastroenterology, Dayanand Medical College and Hospital, Ludhiana, India; 6 Department of Cardiology, Dayanand Medical College and Hospital, Ludhiana, India; 7 Department of Anaesthesia and Critical Care, All India Institute of Medical Sciences, New Delhi, India

**Keywords:** ASCVD, CVD (cardio vascular disease), hepatologic, MASLD, sarcopenia, stroke

## Abstract

**Background and aim:**

Sarcopenia is increasingly recognized as a clinically relevant comorbidity in metabolic dysfunction–associated steatotic liver disease (MASLD), yet its epidemiology and prognostic implications in non-cirrhotic disease remain incompletely characterized. We aimed to evaluate temporal trends in the incidence and prevalence of sarcopenia among patients with MASLD and to assess its association with hepatic and cardiovascular outcomes.

**Methods:**

We conducted a retrospective longitudinal cohort study of adults with MASLD diagnosed between 2005 and 2024 using a large, nationwide electronic health record network. Annual incidence and prevalence of sarcopenia were examined over time. Non-cirrhotic MASLD patients with sarcopenia were compared with non-sarcopenic controls using 1:1 propensity score matching to balance demographic characteristics, metabolic comorbidities, cardiovascular risk factors, and medication use. Hepatic and cardiovascular outcomes were evaluated using Kaplan–Meier analyses and Cox proportional hazards models.

**Results:**

Among 1,232,337 patients with MASLD, 714 had documented sarcopenia. Both incidence and prevalence of sarcopenia increased steadily over the study period, with comparable trends across sexes. After propensity score matching, sarcopenia was associated with significantly higher odds of cirrhosis (OR 4.46), hepatic decompensation (OR 5.18), and all-cause mortality (OR 2.36). Sarcopenic MASLD patients also demonstrated increased risk of major adverse cardiovascular events, including heart failure, ischemic heart disease, atrial fibrillation, cerebrovascular events, and thrombotic disorders. On time-to-event analysis, sarcopenia independently predicted hepatic outcomes (HR 5.03) and cardiovascular outcomes (HR 2.24).

**Conclusion:**

Sarcopenia is an increasingly prevalent condition in MASLD and a powerful independent predictor of adverse hepatic and cardiovascular outcomes in non-cirrhotic disease. These findings underscore the importance of early identification and targeted management of sarcopenia to improve long-term outcomes in MASLD.

## What you need to know

### Background and context

Sarcopenia is increasingly recognized in metabolic dysfunction–associated steatotic liver disease (MASLD), but its epidemiology and prognostic significance in non-cirrhotic populations remain incompletely characterized.

### New findings

In a large nationwide cohort, sarcopenia prevalence increased over time and was independently associated with higher risks of hepatic decompensation, mortality, and major adverse cardiovascular events.

### Impact on patient care

Sarcopenia identifies a high-risk phenotype in MASLD prior to cirrhosis. Routine assessment of muscle health may enhance risk stratification and support integration of exercise and nutritional interventions to improve hepatic and cardiovascular outcomes.

## Introduction

Metabolic dysfunction–associated steatotic liver disease (MASLD) is currently the most prevalent chronic liver disease globally, affecting one in four adults (1). MASLD is a ubiquitous condition affecting persons across all strata, ethnicities, and regions with prevalence rising sharply since the dawn of the 21st century (2). The surge is driven by the rising tide of obesity, type 2 diabetes, and sedentary lifestyles (3, 4). It encompasses a broad spectrum from benign steatosis to hepatitis to advanced fibrosis (5). As the MASLD epidemic intensifies, clinicians face the dual challenge of addressing progressive liver pathology alongside its systemic complications and comorbidities (3). Moreover, cardiovascular disease (CVD) continues to be the leading cause of mortality in MASLD (6, 7). Thus underscoring that MASLD is not only highly prevalent but also clinically consequential, warranting urgent attention from a multisystemic perspective.

Concurrently, sarcopenia, a progressive muscle disease characterized by loss of skeletal muscle mass and strength, has emerged as an important, yet underappreciated, factor intertwined with MASLD (8). Though traditionally and metabolic abnormalities, and manifest in younger adults (9). The revised 2018 criteria of the linked to aging, it is now understood to result from chronic diseases European Working Group on Sarcopenia in Older People (EWGSOP2) and Asian Working Group (AWGS) 2019 emphasize muscle strength as the principal determinant, given its closer association with adverse outcomes compared to muscle mass alone (10). Importantly, sarcopenia is not merely physiologic declined but a pathological state driven by systemic illnesses and lifestyle (11, 12).

Mechanistically, MASLD and sarcopenia are deeply entwined. Skeletal muscle is essential for insulin-mediated glucose uptake; its depletion exacerbates insulin resistance, lipotoxicity, and systemic inflammation, hallmarks of MASLD. Conversely, hepatic steatosis impairs protein metabolism and increases myokine dysregulation, perpetuates chronic inflammation and mitochondrial dysfunction, accelerating muscle loss (13, 14). This bidirectional interplay perpetuates a cycle of metabolic deterioration affecting both liver and muscle.

Clinical data affirm this cross-link as studies have consistently shown that sarcopenia increases the risk of incident MASLD, predicts more severe steatohepatitis, and is independently associated with advanced fibrosis, irrespective of obesity or diabetes. Beyond the liver, sarcopenia portends worse outcomes in metabolic and cardiovascular health, adding to the overall morbidity burden of MASLD patients (15). While its prognostic impact is well-established in cirrhosis, emerging evidence suggests that adverse outcomes begin much earlier (9). Thus, identifying sarcopenia in non-cirrhotic MASLD may provide an opportunity for early intervention through structured exercise and nutritional strategies and alter the outcomes (9).

Despite its clinical relevance and increasing recognition of sarcopenia and MASLD, significant knowledge gaps still persist. Most epidemiological studies to date have been cross-sectional or region-specific, and few have examined long-term outcome trajectories in sarcopenic vs. non-sarcopenic non-cirrhotic MASLD on a large scale. Likewise, the combined impact of sarcopenia on cardiovascular and hepatic outcomes in these subpopulations is not fully elucidated. There is a pressing need for large, longitudinal cohort analyses to elucidate the prevalence trends of sarcopenic non-cirrhotic MASLD, its prognostic implications, and the interplay of risk factors that drive cardiovascular and liver-related outcomes. The current nationwide study intends to address this gap by stratifying MASLD patients into sarcopenic and non-sarcopenic. Through this comprehensive analysis, the current study aims to provide updated real-world evidence to inform risk stratification and management of MASLD, an increasingly recognized but still under-characterized subset of fatty liver disease.

## Methods

### Study design and data source

This was a retrospective, longitudinal cohort study conducted using the U.S. Collaborative Network within TriNetX (Cambridge, MA, USA), a global multi-institutional, federated research network. TriNetX provides real-time access to de-identified electronic health records from over 110 million patients across 66 healthcare organizations in the United States, predominantly comprising large academic medical centers with both inpatient and outpatient facilities. The database captures the full patient population of participating institutions. Data de-identification is performed at the network level and certified through formal expert determination in accordance with the Health Insurance Portability and Accountability Act (HIPAA) Privacy Rule, with additional obfuscation of patient counts fewer than 10 to ensure anonymity. Clinical variables are derived directly from electronic health records of included health care organizations as well as retrieved through a built-in natural language processing system that extracts variables from clinical documents. Robust quality assurance is achieved at the time of extraction from electronic health records before inclusion in the database, in a systematic and standardised format. The process also includes data cleaning which rejects patient records that do not meet the TriNetX quality standards. The database does not include claims data or data collected from randomized clinical trials. The database includes patient data regarding demographics, diagnosis, procedures, laboratory values, and medications. The interface only provides aggregate counts and statistical summaries to protect patient health information and ensures that the data remain de-identified at all levels of data retrieval and dissemination.

### Study population

Adult patients aged 18 years or older with a documented diagnosis of MASLD between January 1, 2005, and December 31, 2024, were eligible for inclusion. MASLD was identified using International Classification of Diseases, Tenth Revision, Clinical Modification (ICD-10-CM) codes for fatty (change of) liver, not elsewhere classified (K76.0), and non-alcoholic steatohepatitis (K75.81) (Supplementary Table S1). The first recorded MASLD diagnosis meeting all eligibility criteria was designated as the cohort entry point. To enhance etiologic specificity, patients with competing causes of chronic liver disease were excluded, including viral hepatitis (B15–B19), alcoholic liver disease (K70), toxic liver disease (K71), autoimmune hepatitis (K75.4), granulomatous hepatitis (K75.3), liver abscess and sequelae of chronic liver disease (ICD-9 572), Wilson disease (E83.01), hemochromatosis (E83.11), alpha-1 antitrypsin deficiency (E88.01), and other specified inflammatory, vascular, or infiltrative liver diseases (K75.0, K75.1, K75.2, K75.89, K76.1–K76.4, and K77) before cohort entry were excluded from outcome-specific analyses to ensure capture of incident events.

### Exposure definitions: sarcopenia

Sarcopenia was defined using ICD-10-CM code M62.84. Patients with MASLD were categorized into sarcopenia and non-sarcopenia cohorts based on the presence or absence of this diagnosis in their longitudinal medical record, with sarcopenia required to be documented on or before the index date (16).

### Index date and follow-up

The index date was defined as the first qualifying MASLD diagnosis that satisfied all inclusion and exclusion criteria. Follow-up commenced one day after the index date to minimize immortal time bias. Patients were followed longitudinally until the occurrence of the outcome of interest, death, loss to follow-up, or the end of available data, whichever occurred first. In accordance with TriNetX platform constraints, patients whose index event occurred more than 20 years prior to analysis were excluded.

### Outcomes

The primary outcome included the incidence of sarcopenia in non-cirrhotic MASLD. Secondary outcomes included assessment of the risk of cardiovascular and hepatic outcomes in non-cirrhotic sarcopenic MASLD cohort compared to non-cirrhotic non-sarcopenic MASLD cohort. Hepatic decompensation was defined as a composite outcome including cirrhosis (K74.6), ascites (R18), esophageal varices (I85), gastric varices (I86.4), hepatic encephalopathy (K76.82), spontaneous bacterial peritonitis (K65.2), hepatorenal syndrome (K76.7), and hepatopulmonary syndrome (K76.81). Mortality was identified using TriNetX demographic death indicators and ICD-10-CM code R99. Cardiovascular outcomes were assessed separately and included major adverse cardiovascular events and related diagnoses, such as acute myocardial infarction (I21, I22), ischemic heart disease (I20–I25), heart failure (I50), atrial fibrillation or flutter (I48), cerebrovascular disease (I60–I69, G45), cardiac arrhythmias and arrest (I46, I47, I49), and thromboembolic events (I26, I80–I82, I81). For each outcome, patients with documented evidence of the event prior to the index date were excluded to ensure incident outcome ascertainment.

### Incidence and prevalence analyses

To evaluate the temporal burden of sarcopenia among patients with MASLD, annual incidence and prevalence estimates were generated using the TriNetX Incidence and Prevalence Tool. These analyses were restricted to the period from 2010 through 2024 to reflect contemporary diagnostic and coding practices. Incidence was defined as the number of new sarcopenia diagnoses per calendar year among MASLD patients without prior sarcopenia, while prevalence was defined as the proportion of MASLD patients with an existing sarcopenia diagnosis in a given year.

### Propensity score matching

To mitigate confounding and balance baseline characteristics between cohorts, 1:1 propensity score matching was performed between MASLD patients with sarcopenia and those without sarcopenia using a greedy nearest-neighbor algorithm with a caliper width of 0.1 pooled standard deviations. Covariates included demographic characteristics, metabolic comorbidities such as obesity, type 2 diabetes mellitus, hypertension, hyperlipidemia, chronic kidney disease, cardiovascular comorbidities, smoking or nicotine dependence, medication use including antihypertensive agents, lipid-lowering therapies, beta-blockers, diuretics, and renin–angiotensin system inhibitors, as well as baseline laboratory values when available. Covariate balance before and after matching was assessed using standardized mean differences, with values below 0.1 indicating adequate balance.

### Statistical analysis

All analyses were performed using the TriNetX browser-based real-time analytics platform (TriNetX Live; TriNetX LLC, Cambridge, MA). Baseline characteristics were summarized using means with standard deviations for continuous variables and proportions for categorical variables. Pre-specified covariates included demographics, metabolic comorbidities, cardiovascular risk factors, smoking status, medication exposures, and relevant laboratory parameters when available.

To minimize confounding, 1:1 propensity score matching was conducted between MASLD patients with and without sarcopenia. Propensity scores were estimated using logistic regression incorporating age, sex, race and ethnicity, obesity, type 2 diabetes, hypertension, dyslipidemia, chronic kidney disease, baseline cardiovascular disease, smoking status, and cardiometabolic medication use. The TriNetX platform applied greedy nearest-neighbor matching with a caliper width of 0.1 pooled standard deviations and randomized observation order to reduce matching bias. Covariate balance was assessed using standardized mean differences, with values <0.1 indicating adequate balance. Separate propensity score models were constructed for hepatic and cardiovascular outcome cohorts, reflecting differences in eligibility criteria and baseline populations

After matching, time-to-event outcomes were evaluated using Kaplan–Meier methods with log-rank testing. Hazard ratios with 95% confidence intervals were estimated using Cox proportional hazards models, and proportionality assumptions were satisfied for all analyses. Continuous variables were compared using the Student's t test. All statistical tests were two-sided, and *p*-values <0.05 were considered statistically significant.

## Results

**Figure 1:**
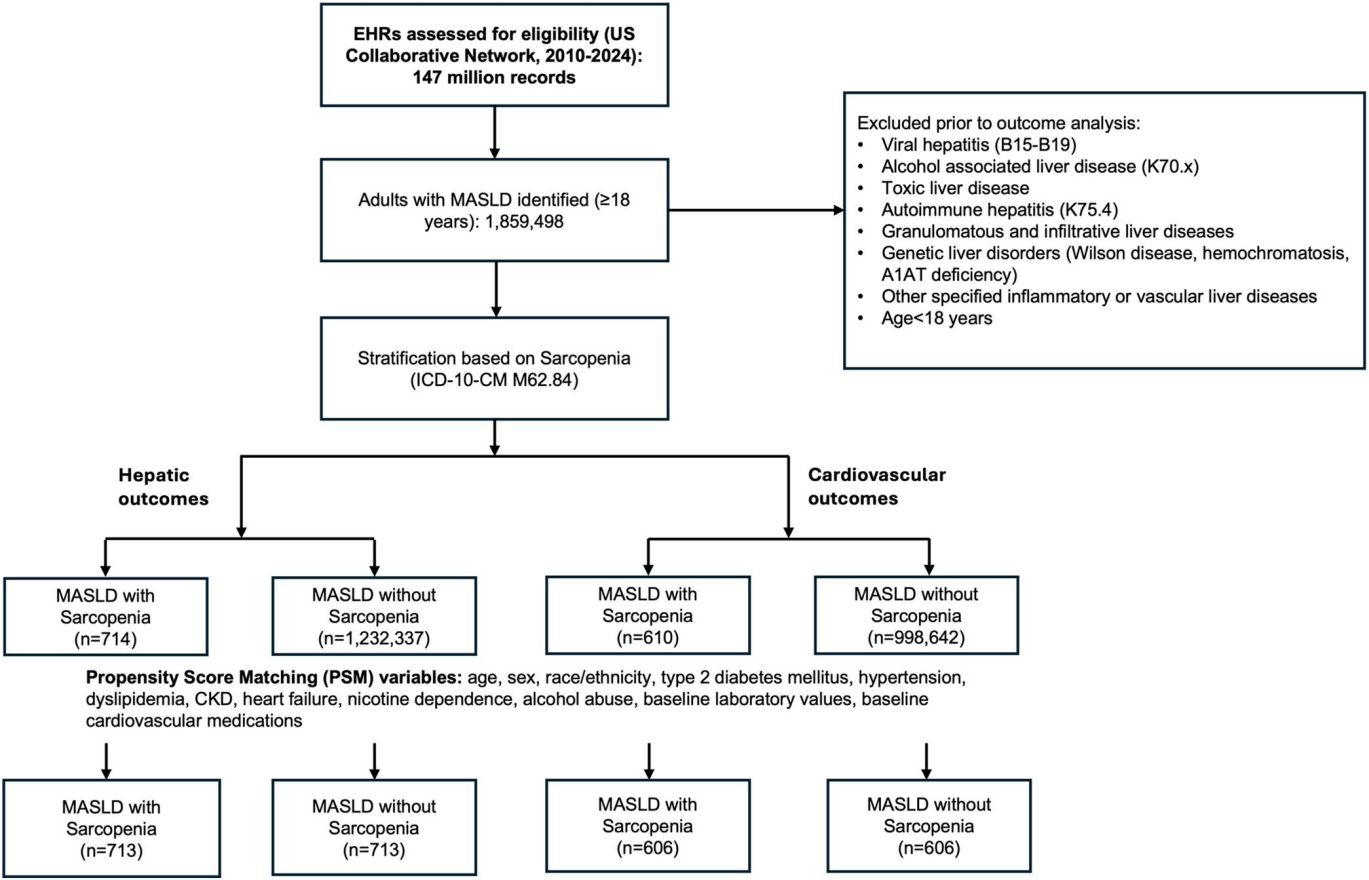
Flow of the study.

**Figure 2:**
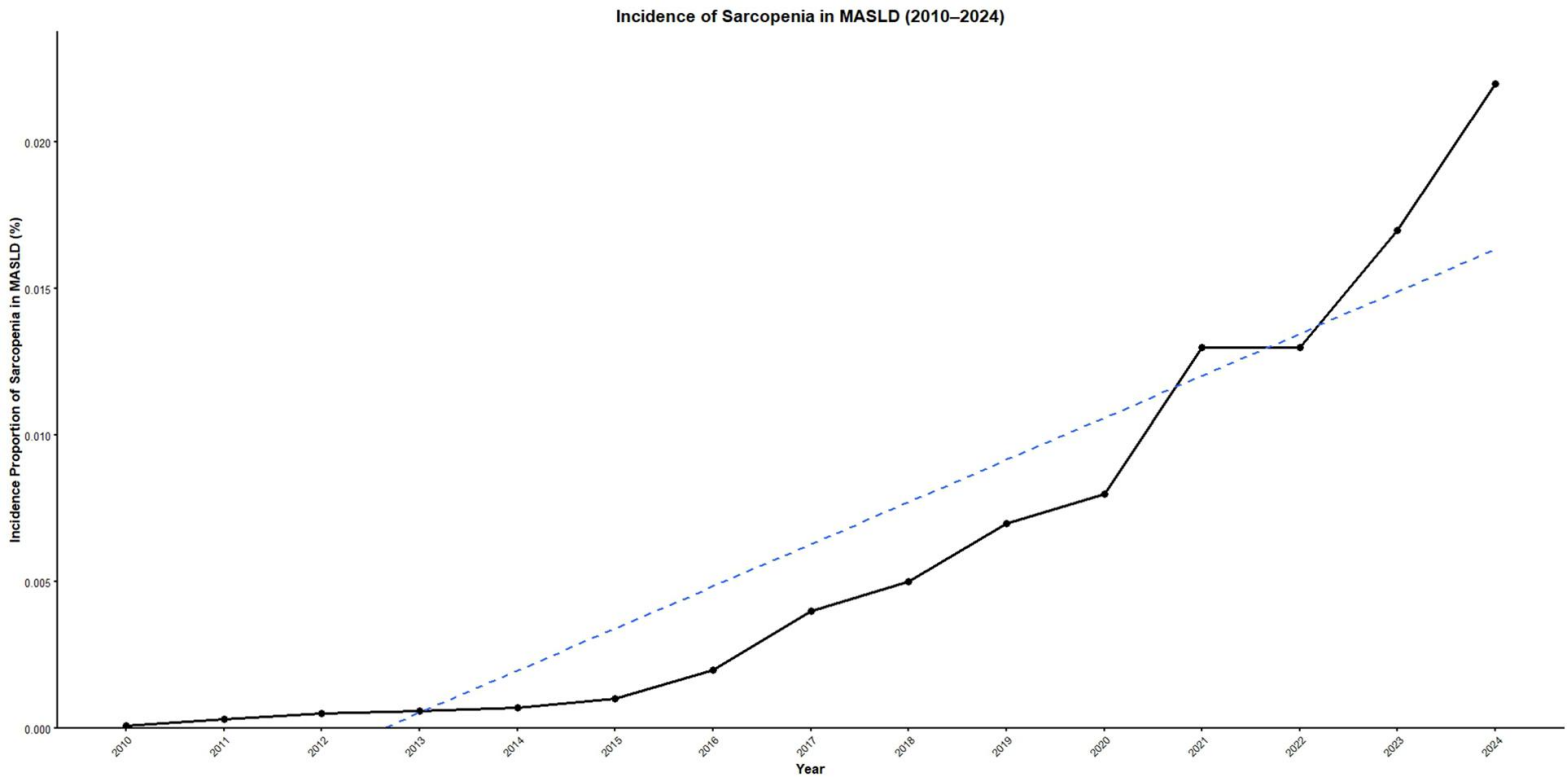
Incidence of sarcopenia in MASLD (2010–2024). Annual incidence proportion of sarcopenia among patients with metabolic dysfunction–associated steatotic liver disease (MASLD) from 2010 to 2024, demonstrating a progressive increase over time; the dashed line represents the linear temporal trend.

**Figure 3:**
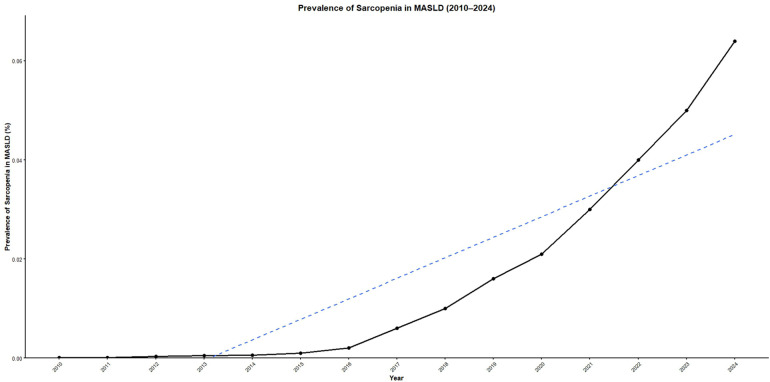
Prevalence of sarcopenia in MASLD (2010–2024). Annual prevalence of sarcopenia among patients with metabolic dysfunction–associated steatotic liver disease (MASLD) from 2010 to 2024, showing a sustained and accelerating increase over the study period; the dashed line represents the linear temporal trend.

A total of 1,859,498 adult patients with a diagnosis of metabolic dysfunction–associated steatotic liver disease (MASLD) were identified within the TriNetX U.S. Collaborative Network during the study period (2015–2024). Among these, 714 patients had a documented diagnosis of sarcopenia (ICD M62.84). Cohort construction and exclusions are detailed in the study flow diagram (Figure 1). The annual incidence proportion of sarcopenia among MASLD patients increased steadily from 0.001% in 2015 to 0.022% in 2024, while prevalence rose from 0.001% to 0.064% over the same period (Figure 2; Supplementary Table S2). Both male and female patients exhibited parallel increases in sarcopenia incidence and prevalence over time. By 2024, prevalence was comparable between sexes, reaching 0.064% in males and 0.065% in females, indicating no substantial sex-based divergence in sarcopenia burden within the MASLD population (Figure 3; Supplementary Table S3).

### Sarcopenia and hepatic outcomes

#### Baseline characteristics for hepatic outcomes

Baseline characteristics for the hepatic outcomes cohort before and after propensity score matching are summarized in Table 1. Before matching, sarcopenic MASLD patients were significantly older and exhibited a higher burden of metabolic comorbidities, cardiovascular disease, chronic kidney disease, and inflammatory markers compared with non-sarcopenic controls. After matching, baseline characteristics were well balanced across cohorts, with standardized mean differences below 0.1 for all included variables.

**Table 1:** Baseline characteristics before and after propensity score matching (for hepatic outcomes).

Characteristics	Before propensity score matching				After propensity score matching			
	MASLD with sarcopenia	MASLD without sarcopenia	*p*-value	SD	MASLD with sarcopenia	MASLD without sarcopenia	*p*-value	SD
N total	714	1,232,337			713	713		
Age at index, (Mean ± SD, years)	65.7 ± 13.5	52.0 ± 15.2	<0.001	0.952	65.7 ± 13.5	65.6 ± 12.1	0.908	0.006
Sex								
Male, N (%)	289	531,083	0.148	0.054	289	289	1	<0.001
Female, N (%)	425	699,135	0.144	0.055	424	423	0.957	0.003
Race/Ethnicity								
White, N (%)	552	913,698	0.061	0.071	551	566	0.335	0.051
Hispanic or Latino, N (%)	65	175,985	<0.001	0.162	65	58	0.509	0.035
Not Hispanic or Latino, N (%)	534	825,672	<0.001	0.170	533	537	0.807	0.013
Black or African American, N (%)	66	98,874	0.235	0.043	66	57	0.396	0.045
Asian, N (%)	28	55,507	0.448	0.029	28	27	0.891	0.007
American Indian or Alaska native, N (%)	10	7,805	0.010	0.076	10	10	1	<0.001
Native Hawaiian or other Pacific Islander, N (%)	10	8,221	0.016	0.072	10	0	0.002	0.100
Comorbidities								
Overweight and Obesity	356	385,238	<0.001	0.385	355	367	0.525	0.034
Type 2 Diabetes Mellitus	340	274,827	<0.001	0.550	339	331	0.671	0.022
Chronic Kidney Disease	203	66,822	<0.001	0.644	202	198	0.814	0.012
Ischemic Heart Diseases	278	129,144	<0.001	0.698	277	279	0.914	0.006
Cerebrovascular Diseases	162	62,209	<0.001	0.528	161	157	0.799	0.013
Human Immunodeficiency Virus (HIV) disease	10	3,639	<0.001	0.121	10	10	1	<0.001
Nicotine dependence	123	137,830	<0.001	0.173	123	129	0.677	0.022
Hyperlipidemia	448	357,464	<0.001	0.719	447	435	0.513	0.035
Laboratory Values								
C reactive protein, (Mean ± SD, mg/dL)	34.1 ± 61.8	20.2 ± 43.2	<0.001	0.385	34.0 ± 61.9	28.7 ± 51.8	0.273	0.092
Hemoglobin A1c, (Mean ± SD, %)	6.4 ± 1.5	6.4 ± 1.6	0.765	0.014	6.4 ± 1.5	6.6 ± 1.6	0.073	0.099
Aspartate Aminotransferase, (Mean ± SD, U/L)	34.4 ± 54.3	34.4 ± 49.0	0.990	<0.001	34.4 ± 54.3	30.0 ± 23.3	0.067	0.106
Alanine Aminotransferase, (Mean ± SD, U/L)	34.2 ± 64.0	44.0 ± 54.1	<0.001	0.166	34.2 ± 64.1	33.3 ± 36.1	0.768	0.017
Cholesterol in HDL, (Mean ± SD, mg/dL)	47.5 ± 17.6	46.2 ± 14.7	0.053	0.081	47.5 ± 17.6	45.8 ± 14.7	0.116	0.103
Cholesterol in LDL, (Mean ± SD, mg/dL)	88.7 ± 40.3	105.3 ± 38.2	<0.001	0.425	88.7 ± 40.3	92.8 ± 42.1	0.122	0.101
Hemoglobin, (Mean ± SD, g/dL)	12.0 ± 2.3	13.7 ± 1.9	<0.001	0.836	12.0 ± 2.3	13.0 ± 2.1	<0.001	0.0479
Platelets, (Mean ± SD, 10*3/uL)	246.0 ± 110.9	258.6 ± 78.5	<0.001	0.131	246.1 ± 111.0	247.9 ± 89.6	0.76	0.018
INR, (Mean ± SD)	443	298,713	<0.001	0.825	1.2 ± 0.4	1.2 ± 0.5	0.565	0.039

INR, international normalized ratio; MASLD, metabolic dysfunction-associated steatotic liver disease; SD, standard deviation.

#### Hepatic outcomes after propensity score matching

**Figure 4:**
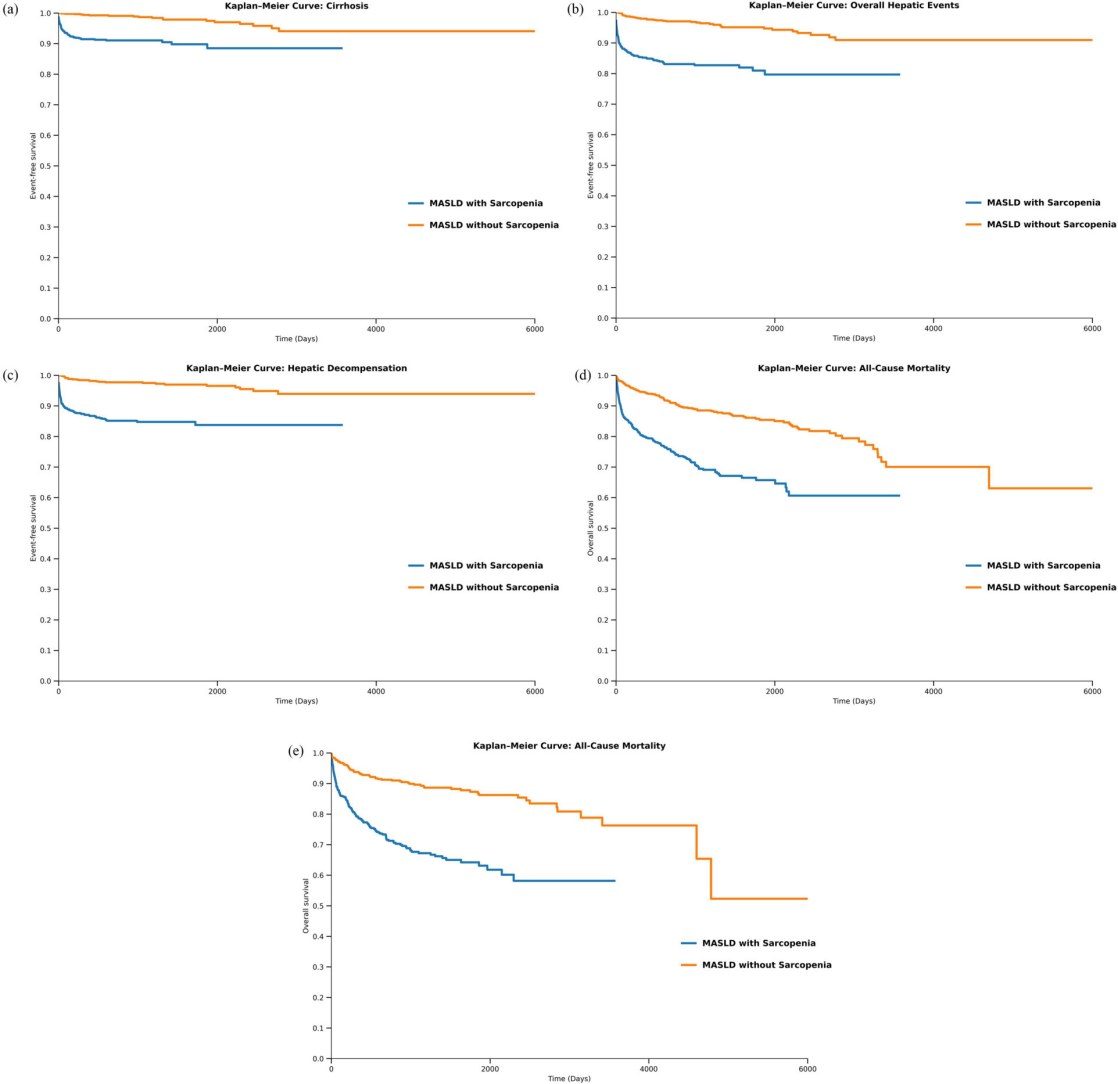
Kaplan–Meier survival analyses comparing patients with metabolic dysfunction–associated steatotic liver disease (MASLD) with sarcopenia vs. MASLD without sarcopenia. Panels depict **(a)** cirrhosis-free survival, **(b)** overall hepatic event–free survival, **(c)** hepatic decompensation–free survival, **(d)** overall survival for hepatic outcomes, and **(e)** overall survival for cardiovascular outcomes. Across all endpoints, patients with sarcopenia demonstrate significantly lower event-free survival and overall survival, with early and sustained separation of survival curves, indicating a higher and earlier risk of adverse hepatic and cardiovascular outcomes compared with non-sarcopenic MASLD patients.

Over a mean follow-up of 757.78 ± 669.04 days in the sarcopenia cohort and 1342.95 ± 1,086.83 days in matched controls, sarcopenic MASLD patients experienced significantly higher rates of adverse hepatic outcomes (Table 2). Cirrhosis developed in 75 sarcopenic MASLD patients compared with 18 non-sarcopenic MASLD patients, corresponding to an OR of 4.46 (95% CI 2.64–7.52). Hepatic decompensation occurred in 120 sarcopenic patients vs. 26 controls (OR 5.18, 95% CI 3.35–8.80). Moreover, all-cause mortality was significantly higher among sarcopenic patients (231 vs. 115 events; OR 2.36, 95% CI 1.85–3.02). Thus demonstrating a marked increase in liver-related morbidity and mortality associated with sarcopenia (Figure 4).

**Table 2:** Hepatic outcomes after propensity score matching.

Outcome	MASLD with sarcopenia	MASLD without sarcopenia	Odds ratio	95% CI
Follow-up time, (Mean ± SD, Days)	757.78 ± 669.04	1,342.95 ± 1,086.83		
Overall Hepatic Events	138	38	4.11	2.84–5.97
Cirrhosis	75	18	4.46	2.64–7.52
Mortality	231	115	2.36	1.85–3.02
Hepatic Decompensation	120	26	5.18	3.35–8.00
Ascites	110	21	5.83	3.62–9.40

CI, confidence interval; MASLD, metabolic dysfunction-associated steatotic liver disease; SD, standard deviation.

#### Predictors of hepatic outcomes

On Cox Proportional analysis, sarcopenia was strongly associated with an increased hazard of adverse hepatic outcomes (HR 5.03, 95% CI 4.21–6.02; *p* < 0.0001) (Table 3). On further Cox Proportional analysis of hepatic outcomes in the sarcopenic MASLD cohort, significant predictors included older age, type 2 diabetes mellitus, elevated HbA1c, lean BMI, and anemia. Higher HDL cholesterol levels, Asian, and African-American ethnicity were associated with lower hepatic risk.

**Table 3:** COX proportionality for hepatic outcomes (MASLD with sarcopenia vs. MASLD without Sarcopenia).

Covariate	Hazard ratio (95% CI)	*p*-value
Sarcopenia	5.03 (4.21–6.02)	<0.0001
Age at Index	1.03 (1.03–1.03)	<0.0001
Male	1.07 (1.05–1.10)	<0.0001
Ethnicity/Race		
Hispanic or Latino	1.14 (1.08–1.19)	<0.0001
Not Hispanic or Latino	1.25 (1.21–1.29)	<0.0001
American Indian or Alaska Native	1.16 (1.00–1.34)	0.0468
Asian	0.59 (0.54–0.64)	<0.0001
Black or African American	0.93 (0.88–0.99)	0.0141
Native Hawaiian or Other Pacific Islander	0.72 (0.61–0.85)	<0.0001
White	1.03 (0.98–1.07)	
BMI		
18–24.9 Kg/m^2^	1.32 (1.27–1.37)	<0.0001
25–29.9 Kg/m^2^	0.95 (0.93–0.98)	0.0013
30–34.9 Kg/m^2^	0.93 (0.90–0.95)	<0.0001
≥35 Kg/m^2^	1.10 (1.07–1.13)	<0.0001
Comorbidities		
Type 2 Diabetes Mellitus	1.26 (1.22–1.30)	<0.0001
Essential Hypertension	0.89 (0.87–0.91)	<0.0001
Hyperlipidemia	0.83 (0.81–0.85)	<0.0001
Chronic Kidney Disease	1.50 (1.44–1.55)	<0.0001
Laboratory values		
Cholesterol in HDL		
≥50 mg/dL	0.75 (0.72–0.77)	<0.0001
Hemoglobin A1c		
≥6.5%	1.12 (1.08–1.17)	<0.0001
Hemoglobin		
<12 g/dL	2.06 (2.01–2.11)	<0.0001

CI, confidence interval; dL, deciliter; g, gram; HDL, high density lipoprotein; Kg, kilogram; LDL, low density lipoprotein; MASLD, metabolic dysfunction-associated steatotic liver disease; mg- milligram).

### Sarcopenia and cardiovascular outcomes

#### Baseline characteristics for cardiovascular outcomes

For cardiovascular outcome analyses, patients with baseline cardiovascular disease or insufficient follow-up were excluded, resulting in a reduced cohort (610 sarcopenic and 998,642 non-sarcopenic MASLD patients) prior to propensity score matching (Table 4). Baseline characteristics for the CV outcomes cohort before and after propensity score matching are summarized in Table 4. Prior to matching, MASLD patients with sarcopenia were significantly older and had a higher prevalence of metabolic comorbidities, cardiovascular disease, chronic kidney disease, and greater use of cardiometabolic medications compared with non-sarcopenic MASLD patients. Laboratory parameters, including inflammatory markers and lipid indices, also differed significantly between groups. After propensity score matching, all baseline demographic, clinical, medication, and laboratory variables were well balanced between cohorts, with standardized mean differences below 0.1 for all covariates, indicating successful matching.

**Table 4:** Baseline characteristics before and after propensity score matching (for cardiovascular outcomes).

Characteristics	Before propensity score matching				After propensity score matching			
	MASLD with sarcopenia	MASLD without sarcopenia	*p*-value	SD	MASLD with sarcopenia	MASLD without sarcopenia	*p*-value	SD
N total	610	998,642			606	606		
Age at index, (Mean ± SD, years)	62.9 ± 12.5	49.6 ± 14.8	<0.001	0.968	62.8 ± 12.6	63.0 ± 11.5	0.792	0.015
Sex								
Male, N (%)	230	418,455	0.033	0.087	227	223	0.812	0.014
Female, N (%)	380	578,350	0.032	0.088	379	383	0.812	0.014
Race/Ethnicity								
White, N (%)	464	726,372	0.074	0.074	461	457	0.789	0.015
Hispanic or Latino, N (%)	82	159,299	0.088	0.072	81	93	0.326	0.056
Not Hispanic or Latino, N (%)	423	645,685	0.018	0.098	420	398	0.177	0.078
Black or African American, N (%)	32	75,256	0.031	0.094	32	33	0.899	0.007
Asian, N (%)	18	48,548	0.028	0.099	18	15	0.596	0.030
American Indian or Alaska native, N (%)	10	6,795	0.004	0.090	10	10	1	<0.001
Native Hawaiian or other Pacific Islander, N (%)	0	6,722	0.042	0.117	0	10	0.001	0.100
Medications								
Antilipemic agents	225	193,422	<0.001	0.396	224	212	0.473	0.041
Antihypertensives	88	60,682	<0.001	0.278	85	89	0.743	0.019
Diuretics	315	156,994	<0.001	0.821	311	304	0.688	0.023
Beta blocking agents	246	145,773	<0.001	0.601	242	251	0.599	0.030
Calcium channel blockers	118	94,507	<0.001	0.284	117	119	0.885	0.008
Agents acting on RAS	205	190,802	<0.001	0.333	203	214	0.506	0.038
Comorbidities								
Overweight and Obesity	224	275,764	<0.001	0.195	222	239	0.314	0.058
Type 2 Diabetes Mellitus	229	173,691	<0.001	0.463	225	227	0.905	0.007
Chronic Kidney Disease	97	28,588	<0.001	0.459	94	90	0.749	0.018
Hypertensive diseases	327	327,120	<0.001	0.429	324	319	0.774	0.017
Human Immunodeficiency Virus (HIV) disease	10	2,816	<0.001	0.139	10	10	1	<0.001
Nicotine dependence	72	89,892	0.016	0.091	71	80	0.434	0.045
Hyperlipidemia	233	212,702	<0.001	0.375	232	231	0.953	0.003
Liver transplant status	15	577	<0.001	0.217	12	11	0.833	0.012
Malignant neoplasm of liver and intrahepatic bile ducts	29	1,780	<0.001	0.298	26	24	0.773	0.017
Laboratory Values								
C reactive protein, (Mean ± SD, mg/dL)	26.0 ± 57.1	17.6 ± 38.7	0.013	0.173	26.0 ± 57.1	24.0 ± 44.7	0.753	0.039
Hemoglobin A1c, (Mean ± SD, %)	6.2 ± 1.7	6.3 ± 1.6	0.497	0.037	6.3 ± 1.8	6.5 ± 1.6	0.085	0.0144
Aspartate Aminotransferase, (Mean ± SD, U/L)	43.2 ± 69.2	35.9 ± 52.8	0.003	0.118	43.1 ± 69.3	37.7 ± 39.7	0.144	0.096
Alanine Aminotransferase, (Mean ± SD, U/L)	35.7 ± 45.9	47.2 ± 60.7	<0.001	0.214	35.3 ± 44.9	40.9 ± 41.2	0.048	0.13
Cholesterol in HDL, (Mean ± SD, mg/dL)	46.9 ± 18.6	46.6 ± 14.7	0.697	0.021	47.1 ± 18.5	47.5 ± 14.2	0.752	0.028
Cholesterol in LDL, (Mean ± SD, mg/dL)	96.1 ± 43.4	109.5 ± 36.7	<0.001	0.333	96.3 ± 43.5	105.4 ± 39.1	0.012	0.22
Hemoglobin, (Mean ± SD, g/dL)	11.8 ± 2.4	13.8 ± 1.8	<0.001	0.935	11.9 ± 2.4	13.2 ± 2.0	<0.001	0.060
Platelets, (Mean ± SD, 10*3/uL)	204.6 ± 111.3	261.6 ± 78.7	<0.001	0.591	204.9 ± 111.3	242.9 ± 96.3	<0.001	0.0523
INR, (Mean ± SD)	1.2 ± 0.3	1.1 ± 0.2	<0.001	0.673	1.2 ± 0.3	1.1 ± 0.2	<0.001	0.0523

dL, deciliter; g, gram; HDL, high density lipoprotein; Kg, kilogram; LDL, low density lipoprotein; INR, international normalized ratio; MASLD, metabolic dysfunction-associated steatotic liver disease; uL, microliter; RAS, renin-angiotensin system; SD, standard deviation.

#### Cardiovascular outcomes after propensity score matching

**Figure 5:**
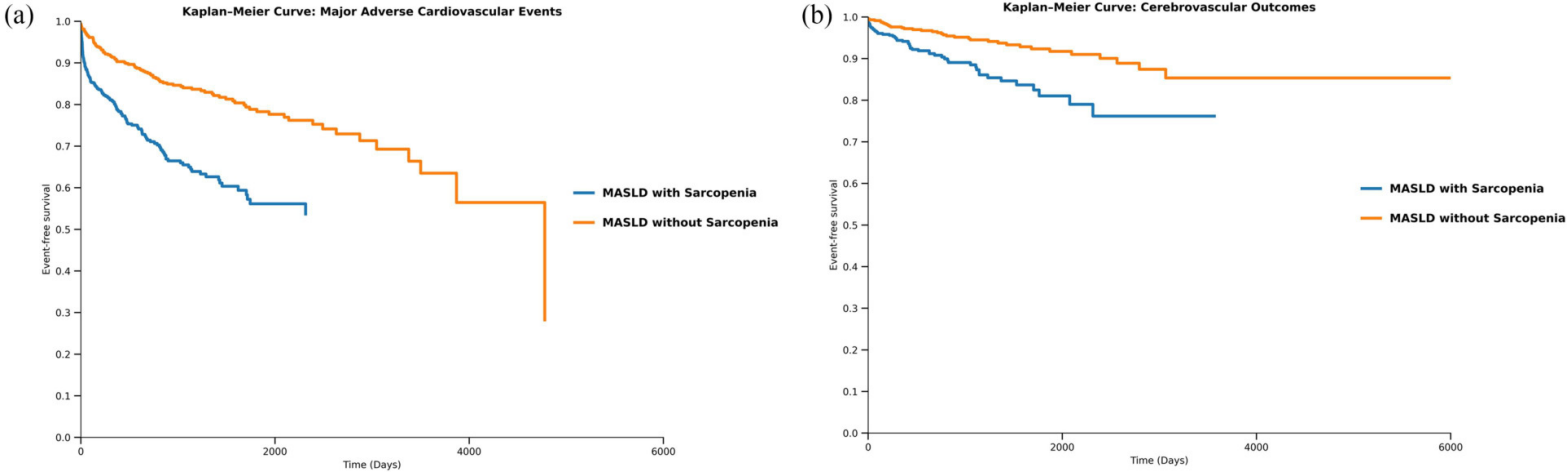
Kaplan–Meier survival analyses comparing patients with metabolic dysfunction–associated steatotic liver disease (MASLD) with sarcopenia vs. MASLD without sarcopenia for cardiovascular outcomes. Panels depict **(a)** major adverse cardiovascular event (MACE)–free survival and **(b)** cerebrovascular event–free survival. Patients with sarcopenia demonstrate significantly lower event-free survival across cardiovascular endpoints, indicating a higher risk of both composite and cerebrovascular events compared with non-sarcopenic MASLD patients.

Following propensity score matching, MASLD patients with sarcopenia demonstrated significantly higher rates of adverse cardiovascular outcomes compared with matched non-sarcopenic controls (Table 5). Over a mean follow-up of 762.5 ± 722.5 days in the sarcopenia cohort and 1,308.1 ± 1,108.9 days in matched controls, MASLD patients with sarcopenia experienced significantly higher rates of adverse CV outcomes (Table 5). All-cause mortality occurred more frequently in sarcopenic patients [166 vs. 69 events; odds ratio (OR) 2.94, 95% CI 2.16–3.99]. Major adverse CV events were also more common in the sarcopenia cohort (162 vs. 103 events; OR 1.78, 95% CI 1.35–2.35). Sarcopenia was associated with increased odds of atrial fibrillation/flutter (OR 1.99, 95% CI 1.18–3.33), ischemic heart disease (OR 1.60, 95% CI 1.17–2.19), and heart failure (OR 2.06, 95% CI 1.34–3.16). Furthermore, significantly increased risk of cerebrovascular events (OR 1.64, 95% CI 1.07–2.54), and thrombotic disorders (OR 2.84, 95% CI 1.77–4.57) was observed in sarcopenic MASLD cohorts (Figure 5).

**Table 5:** Cardiovascular outcomes after propensity score matching.

Outcome	MASLD with sarcopenia	MASLD without sarcopenia	Odds ratio	95% CI
Follow up time, (Mean ± SD, Days)	762.53 ± 722.46	1,308.11 ± 1,108.87		
Mortality	166	69	2.94	2.16–3.99
MACE*	162	103	1.78	1.35–2.35
Atrial fibrillation/flutter	44	23	1.99	1.18–3.33
Ischemic Heart Disease	113	76	1.60	1.17–2.19
Heart Failure	66	34	2.06	1.34–3.16
Cerebrovascular events	57	36	1.64	1.07–2.54
Thrombotic Disorders	66	25	2.84	1.77–4.57

CI, confidence interval; MACE, major adverse cardiovascular event; MASLD, metabolic dysfunction-associated steatotic liver disease; SD, standard deviation.

*MACE includes non-fatal myocardial infarction, non-fatal stroke, and cardiovascular death.

#### Predictors of cardiovascular outcomes

**Figure 6:**
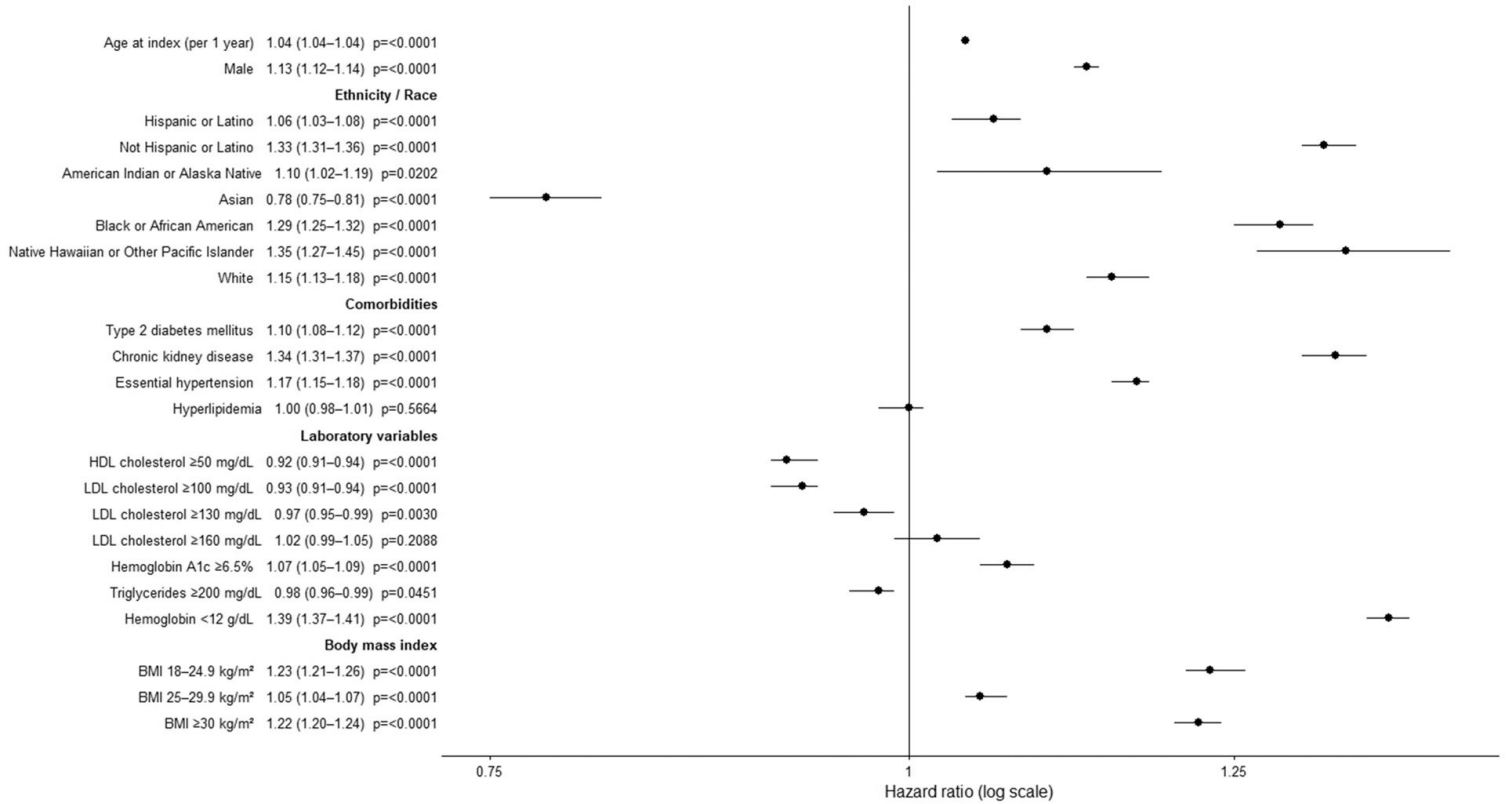
Forest plot of adjusted hazard ratios (HRs) with 95% confidence intervals from the multivariable Cox proportional hazards model for cardiovascular outcomes.

On Cox Proportional analysis, sarcopenia was strongly associated with an increased hazard of adverse cardiovascular outcomes (HR 2.24, 95% CI 1.91–2.62; *p* < 0.0001) (Table 6). On further Cox Proportional analysis of hepatic outcomes in sarcopenic MASLD cohort, increasing age, male sex, type 2 diabetes mellitus, hypertension, elevated HbA1c, lean BMI range (18–24.9 kg/m^2^), and anemia were also associated with higher cardiovascular risk. Higher HDL cholesterol levels and Asian ethnicity were associated with a reduced hazard of cardiovascular outcomes (Figure 6).

**Table 6:** COX proportionality for cardiovascular outcomes (MASLD with sarcopenia vs. MASLD without Sarcopenia).

Covariate	Hazard ratio (95% CI)	*p*-value
MASLD with Sarcopenia or MASLD without Sarcopenia	2.24 (1.91–2.62)	<0.0001
Age at Index	1.04 (1.04–1.04)	<0.0001
Male	1.13 (1.12–1.14)	<0.0001
Ethnicity/Race		
Hispanic or Latino	1.06 (1.03–1.08)	<0.0001
Not Hispanic or Latino	1.33 (1.31–1.36)	<0.0001
American Indian or Alaska Native	1.10 (1.02–1.19)	0.0202
Asian	0.78 (0.75–0.81)	<0.0001
Black or African American	1.29 (1.25–1.32)	<0.0001
Native Hawaiian or Other Pacific Islander	1.35 (1.27–1.45)	<0.0001
White	1.15 (1.13–1.18)	<0.0001
Comorbidities		
Type 2 Diabetes Mellitus	1.10 (1.08–1.12)	<0.0001
Essential Hypertension	1.17 (1.15–1.18)	<0.0001
Laboratory values		
Cholesterol in HDL		
≥50 mg/dL	0.92 (0.91–0.94)	<0.0001
Hemoglobin A1c		
≥6.5%	1.07 (1.05–1.09)	<0.0001
BMI		
18–24.9 Kg/m^2^	1.23 (1.21–1.26)	<0.0001
25–29.9 Kg/m^2^	1.05 (1.04–1.07)	<0.0001
≥30 Kg/m^2^	1.22 (1.20–1.24)	<0.0001
Hemoglobin		
<12 g/dL	1.39 (1.37–1.41)	<0.0001

CI, confidence interval; dL, deciliter; g, gram; HDL, high density lipoprotein; Kg, kilogram; LDL, low density lipoprotein; MASLD, metabolic dysfunction-associated steatotic liver disease; mg, milligram.

## Discussion

The current large, nationwide, real-world cohort study spanning more than a decade demonstrated a steadily rising burden of sarcopenia among MASLD patients, accompanied by a profound and independent association with adverse hepatic and cardiovascular outcomes. Using robust propensity-matched analyses, sarcopenia emerged as a powerful predictor of cirrhosis, hepatic decompensation, and all-cause mortality, conferring more than five-fold higher hazards for hepatic events and nearly three-fold higher odds of death. Importantly, sarcopenia was also independently associated with a broad spectrum of cardiovascular complications, including major adverse cardiovascular events, heart failure, cerebrovascular disease, and thrombotic disorders, underscoring its role as a systemic risk modifier rather than a mere musculoskeletal comorbidity. These findings position sarcopenia as a critical co-factor in MASLD prognosis.

Our results align with a growing body of literature linking low muscle mass i.e., sarcopenia to worse clinical outcomes in MASLD. Prior cohort studies have shown that MASLD patients with sarcopenia are significantly more prone to advanced liver disease (17). A study demonstrated that sarcopenia doubled the odds of significant fibrosis, independent of obesity and insulin resistance (18). Similarly, a nationwide analysis ∼1.8-fold higher odds of significant fibrosis in sarcopenic MASLD patients, an association that persisted across BMI categories and after adjusting for metabolic confounders (19). This was further concluded by a recent meta-analysis with sarcopenic MASLD patients have pooled OR ∼1.5 of advanced fibrosis than those without sarcopenia (20). While the prognostic impact of sarcopenia in cirrhosis is well established (predicting higher mortality and complications), our study extends this risk to non-cirrhotic populations, underscoring that adverse outcomes begin to accrue at earlier disease stages (21). Perhaps the most novel aspect of our findings is the strong link between sarcopenia and cardiovascular events in MASLD. Cardiovascular disease is the leading cause of mortality in MASLD, and emerging evidence suggests that coexisting sarcopenia exacerbates this risk (15, 21). Sarcopenia was associated with a 320% increase in cardiovascular mortality among individuals with MASLD (21). Studies also reported roughly four-fold higher odds of an elevated 10-year ASCVD risk score in sarcopenic MASLD than metabolically healthy controls, and in patients with type 2 diabetes, sarcopenic MASLD was significantly associated with accelerated carotid atherosclerosis progression (adjusted OR ∼2.2 over 6–8 years) (22, 23). Taken together, these data highlight a synergistic interaction between poor muscle status and fatty liver in driving both liver-related and cardiovascular morbidity. Notably, most prior studies were cross-sectional or included patients with advanced fibrosis, whereas our cohort was limited to non-cirrhotic MASLD; this strengthens the evidence that sarcopenia confers excess risk even before cirrhosis has developed.

**Figure 7:**
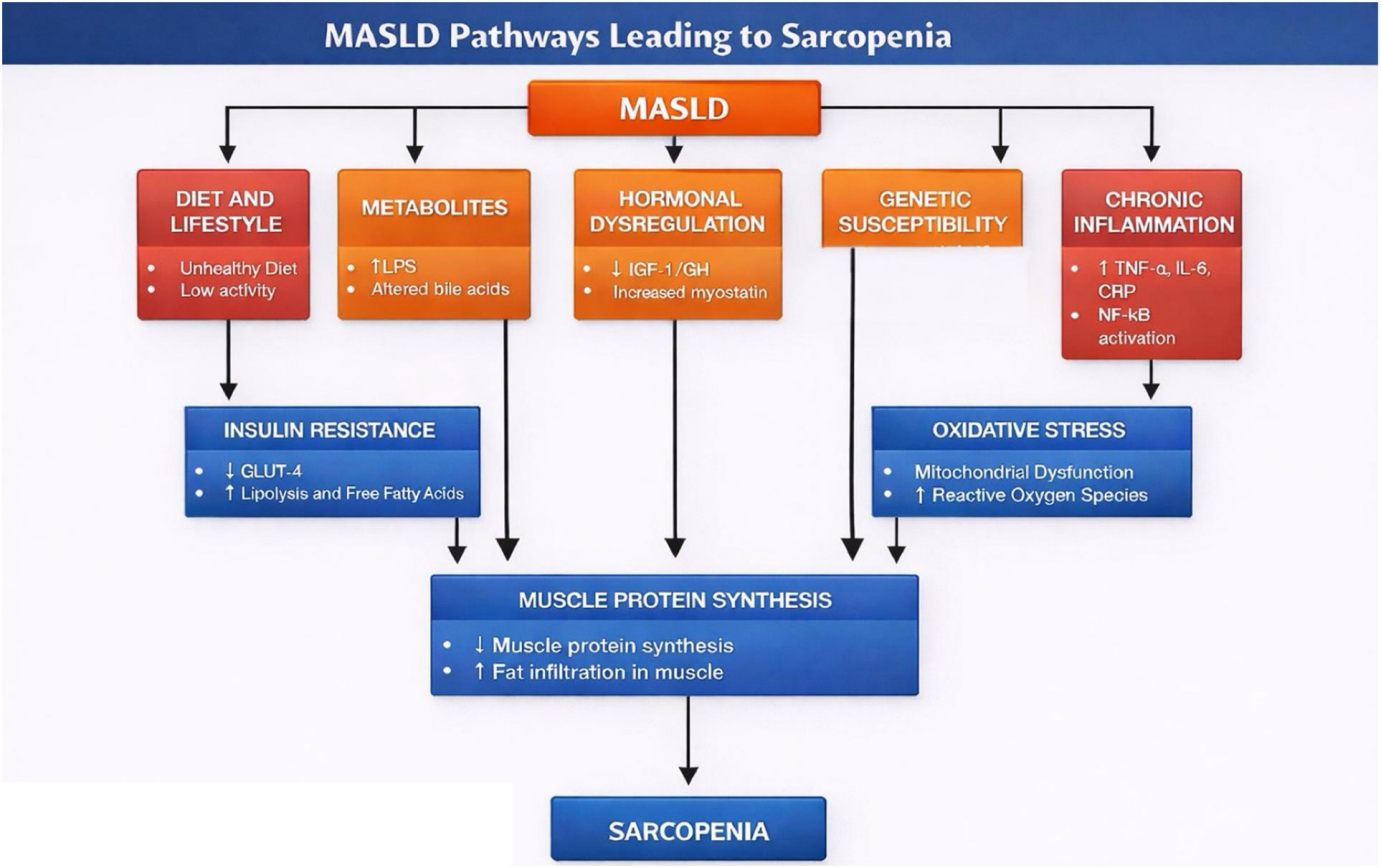
Pathways leading to sarcopenia in MASLD. (CRP, C reactive protein; GF, growth factor; GLUT, glucose transporter, IGF, insulin like growth factor; IL-interleukin; LPS, Lipopolysaccharide; MASLD, metabolic dysfunction-associated live disease; NF-nuclear factor; TNF, Tumor necrosis factor).

**Figure 8:**
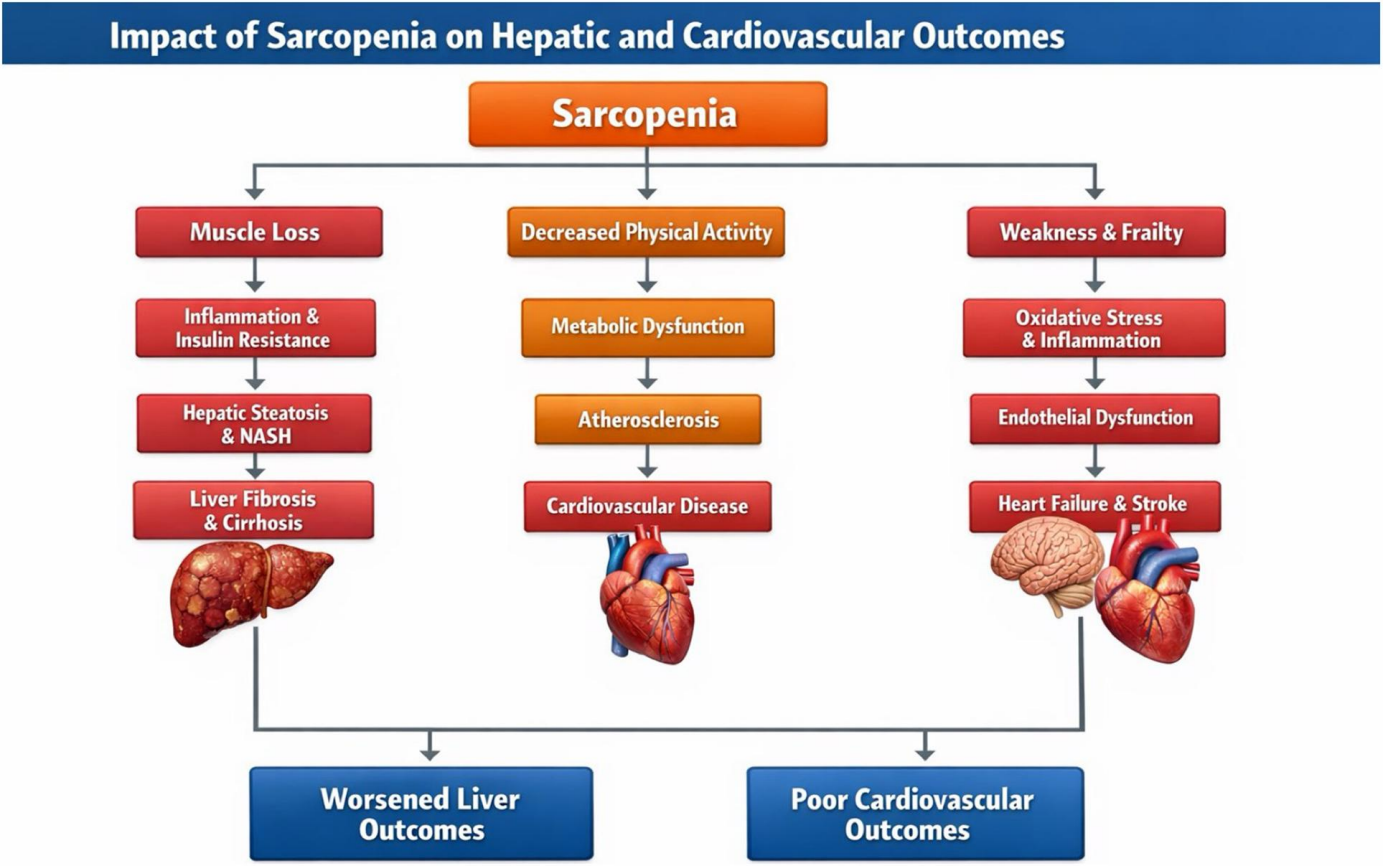
Impact of sarcopenia on hepatic and cardiovascular outcomes in MASLD. (NASH, non-alcoholic steatohepatitis)

Several biological mechanisms plausibly underlie the observed bidirectional link between sarcopenia and MASLD (Figures 7, 8). Chronic low-grade inflammation is a central driver: excess visceral adiposity in MASLD releases pro-inflammatory cytokines such as TNF-α and IL-6, which can trigger muscle protein catabolism and functional decline (24). Hepatic steatosis itself contributes to systemic inflammation and disrupts protein metabolism, leading to myocyte mitochondrial dysfunction and accelerated muscle loss (25). Reciprocally, loss of skeletal muscle mass exacerbates insulin resistance—since muscle is the principal site of insulin-mediated glucose disposal—thereby promoting hyperglycemia and increased hepatic lipogenesis (26). This creates a vicious cycle where sarcopenia worsens the metabolic milieu that drives steatohepatitis. Hormonal dysregulation is also implicated: patients with metabolic syndrome often exhibit reduced growth hormone/IGF-1 and testosterone levels, which impairs muscle anabolism and may exacerbate hepatic fat accumulation (27). At the same time, the fatty liver secretes hepatokines (e.g., fetuin-A) and induces hyperinsulinemia, both of which can further promote muscle wasting, while the loss of myokines from muscle (such as irisin or IL-15) removes signals that normally help attenuate liver fat and inflammation (28). Thus, inflammation, insulin resistance, and endocrine alterations form an integrated muscle–liver cross-talk network that fuels both sarcopenia and MASLD progression in tandem (29, 30).

The current study has several strengths, including the large sample size drawn from a nationwide multi-center database and a long observation period of up to 15 years. The use of a real-world EHR network (TriNetX) enhances generalizability, capturing a diverse MASLD population across academic and community settings. The study rigorously excluded other chronic liver diseases and applied propensity score matching to balance key confounders, lending credibility to the observed associations. Nevertheless, important limitations must be acknowledged. First, our exposure and outcomes were ascertained via ICD codes, which may be subject to misclassification. Sarcopenia in particular is likely underdiagnosed and under-coded in practice, meaning our “sarcopenia” cohort probably represents more severe cases where the condition was recognized clinically. As a result, some MASLD patients with mild to moderate muscle loss might have been misclassified in the non-sarcopenia group, potentially biasing our estimates toward the null. Moreover, the ICD code for sarcopenia does not capture muscle function or strength. We lacked direct measurements of muscle mass or performance (e.g., DEXA scans, CT muscle area, grip strength), which are now considered the gold-standard for sarcopenia assessment. Additionally, there may be selection bias in healthcare utilization: patients with more health issues (either sarcopenia or metabolic comorbidities) might undergo more frequent visits and thus have more opportunities for outcome detection. The median follow-up was shorter in the sarcopenia group than in controls, partly due to higher mortality, which might affect the observed incidence rates. Finally, as an observational study, our findings show association but cannot prove causation. It remains possible that more aggressive MASLD drives muscle loss (reverse causality) or that a third factor (e.g., systemic inflammation burden) independently worsens both muscle and liver health.

From a clinical perspective, our findings have important implications and highlight avenues for future research. They underscore the need to screen for sarcopenia in patients with MASLD—particularly older individuals and those with coexisting metabolic disease—even before the onset of cirrhosis. Simple assessments such as grip strength, gait speed, or body composition analysis could be feasibly incorporated into routine MASLD clinics to enable early detection. This risk stratification is clinically relevant, as emerging evidence suggests that sarcopenia identifies patients at higher risk of fibrosis progression and cardiovascular events (23).

Importantly, sarcopenia is a modifiable risk factor. Unlike fixed genetic determinants, muscle mass and function can be improved through targeted interventions. Lifestyle modification remains the cornerstone of management, with structured exercise—particularly resistance training combined with aerobic activity—and adequate protein intake shown to improve muscle strength and mass while also conferring metabolic and hepatic benefits, independent of weight loss. Our findings support integrating exercise physiology services and nutritional counselling into MASLD care, thereby addressing both liver-related outcomes and cardiovascular risk.

Although several pharmacologic strategies aimed at muscle preservation such as anabolic agents (testosterone, GH supplement), myostatin inhibitors, and selective androgen receptor modulators, etc. are not currently approved for sarcopenia in MASLD, and lifestyle interventions remain the most evidence-based approach. Looking ahead, prospective studies and randomized trials are needed to determine whether targeted treatment of sarcopenia can translate into reductions in cirrhosis and cardiovascular events. Incorporating quantitative muscle assessments into MASLD risk models may further refine prognostication. Overall, our findings support a more integrated approach to MASLD management that recognizes muscle health as a key determinant of long-term outcomes.

## Data Availability

The original contributions presented in the study are included in the article/Supplementary Material, further inquiries can be directed to the corresponding author.
