# Supplementary material for: Incidence, prevalence, and prognostic impact of sarcopenia on hepatic and cardiovascular outcomes in non-cirrhotic metabolic dysfunction–associated steatotic liver disease
Source: Front Epidemiol. 2026 Jun 11;6:1779600. doi: 10.3389/fepid.2026.1779600 (PMC13294098; doi:10.3389/fepid.2026.1779600)
Supplement: Supplementary file 1 [file Table1.docx]

**Table 1 – ICD Codes used in the study**

| ICD Code | Clinical Description |
| --- | --- |
| K76.0 | Fatty (change of) liver, not elsewhere classified |
| K75.81 | Nonalcoholic steatohepatitis (NASH) |
| M62.84 | Sarcopenia |
| F10 | Alcohol related disorders |
| I21 | Acute myocardial infarction |
| I22 | Subsequent myocardial infarction |
| I26 | Pulmonary embolism |
| I30 | Acute pericarditis |
| I31 | Other diseases of pericardium |
| I32 | Pericarditis in diseases classified elsewhere |
| I40 | Acute myocarditis |
| I41 | Myocarditis in diseases classified elsewhere |
| I46 | Cardiac arrest |
| I47.0 | Re-entry ventricular arrhythmia |
| I47.2 | Ventricular tachycardia |
| I48 | Atrial fibrillation and flutter |
| I49 | Other cardiac arrhythmias |
| I49.3 | Ventricular premature depolarization |
| I50 | Heart failure |
| I60–I69 | Cerebrovascular diseases |
| G45 | Transient cerebral ischemic attacks |
| I80 | Phlebitis and thrombophlebitis |
| I81 | Portal vein thrombosis |
| I82 | Other venous embolism and thrombosis |
| K70 | Alcoholic liver disease |
| K71 | Toxic liver disease |
| K72 | Hepatic failure |
| K73 | Chronic hepatitis |
| K74.0 | Hepatic fibrosis |
| K74.1 | Hepatic sclerosis |
| K74.2 | Hepatic fibrosis with sclerosis |
| K74.3 | Primary biliary cirrhosis |
| K74.4 | Secondary biliary cirrhosis |
| K74.5 | Biliary cirrhosis, unspecified |
| K74.6 | Other and unspecified cirrhosis of liver |
| K75.0 | Abscess of liver |
| K75.1 | Phlebitis of portal vein |
| K75.2 | Nonspecific reactive hepatitis |
| K75.3 | Granulomatous hepatitis |
| K75.4 | Autoimmune hepatitis |
| K75.9 | Inflammatory liver disease, unspecified |
| K75.89 | Other specified inflammatory liver diseases |
| K76.1 | Chronic passive congestion of liver |
| K76.2 | Central hemorrhagic necrosis of liver |
| K76.3 | Infarction of liver |
| K76.4 | Peliosis hepatis |
| K76.5 | Hepatic veno-occlusive disease |
| K76.6 | Portal hypertension |
| K76.7 | Hepatorenal syndrome |
| K76.81 | Hepatopulmonary syndrome |
| K76.82 | Hepatic encephalopathy |
| K77 | Liver disorders in diseases classified elsewhere |
| R18 | Ascites |
| K65.2 | Spontaneous bacterial peritonitis |
| I85 | Esophageal varices |
| I86.4 | Gastric varices |
| C22.0 | Liver cell carcinoma |
| C22.8 | Malignant neoplasm of liver, primary, unspecified |
| E88.01 | Alpha-1-antitrypsin deficiency |
| E83.01 | Wilson disease |
| E83.11 | Hemochromatosis |
| R99 | Ill-defined and unknown cause of mortality |
| Z94.4 | Liver transplant status |
| T86.4 | Complications of liver transplant |
| B15–B19 | Viral hepatitis |
| 571 | Chronic liver disease and cirrhosis (ICD-9) |
| 572 | Liver abscess and sequelae of chronic liver disease (ICD-9) |
| 573 | Other disorders of liver (ICD-9) |

**Table 2 - Overall Incidence Proportion and Prevalence of Sarcopenia**

| **Year** | **Incidence Proportion** | **Prevalence** |
| --- | --- | --- |
| 2015 | 0.001% (10) | 0.001% (10) |
| 2016 | 0.002% (20) | 0.002% (22) |
| 2017 | 0.004% (57) | 0.006% (78) |
| 2018 | 0.005% (73) | 0.010% (141) |
| 2019 | 0.007% (103) | 0.016% (231) |
| 2020 | 0.008% (114) | 0.021% (315) |
| 2021 | 0.013% (194) | 0.030% (457) |
| 2022 | 0.013% (198) | 0.040% (602) |
| 2023 | 0.017% (248) | 0.050% (734) |
| 2024 | 0.022% (298) | 0.064% (875) |

**Table 3 - Sex Stratified Incidence Proportion and Prevalence of Sarcopenia**

| **Year** | **Incidence Proportion** | | **Prevalence** | |
| --- | --- | --- | --- | --- |
|  | **Male** | **Female** | **Male** | **Female** |
| 2015 | 0.002 % (10) | 0.001% (10) | 0.002% (10) | 0.001% (10) |
| 2016 | 0.002 % (10) | 0.002% (12) | 0.002% (10) | 0.001% (13) |
| 2017 | 0.004 % (21) | 0.005% (36) | 0.005% (30) | 0.006% (48) |
| 2018 | 0.005 % (33) | 0.005% (40) | 0.009% (54) | 0.011% (87) |
| 2019 | 0.006 % (42) | 0.007% (61) | 0.013% (87) | 0.017% (144) |
| 2020 | 0.007 % (49) | 0.008% (65) | 0.019% (125) | 0.023% (190) |
| 2021 | 0.015 % (104) | 0.011% (90) | 0.030% (204) | 0.030% (253) |
| 2022 | 0.015 % (100) | 0.012% (98) | 0.041% (274) | 0.039% (328) |
| 2023 | 0.018 % (118) | 0.016% (130) | 0.051% (332) | 0.050% (402) |
| 2024 | 0.022 % (130) | 0.022% (168) | 0.064% (382) | 0.065% (493) |
